# Evaluating the Impact of Telehealth Prenatal Care at a Federally Qualified Health Center

**DOI:** 10.1111/jmwh.70107

**Published:** 2026-05-21

**Authors:** Catherine Daily, Ashley Gresh, Elizabeth R. Hamilton, Nancy A. Crowell, Germán Gustavo Alva Martinez, Christina X. Marea

**Affiliations:** ^1^ Georgetown University School of Nursing Washington District of Columbia; ^2^ Mary's Center Washington District of Columbia; ^3^ Johns Hopkins University School of Nursing Baltimore Maryland; ^4^ City and County of San Francisco Department of Public Health San Francisco California

**Keywords:** antepartum care, community health services, community health, group prenatal care/CenteringPregnancy, health equity, midwives, patient education, patient satisfaction, prenatal care, telehealth

## Abstract

**Introduction:**

During the COVID‐19 pandemic, clinicians had to reimagine perinatal care. Mary's Center, a federally qualified health center, built an innovative telehealth group prenatal care (T‐GPNC) program to ensure equitable and safe access to health care. T‐GPNC includes an individual telehealth assessment and group education. We conducted a program evaluation to determine the adequacy of prenatal care for T‐GPNC clients compared with individual prenatal care (IPC) clients and to describe client satisfaction with T‐GPNC.

**Methods:**

This mixed‐methods study included a retrospective chart review of electronic health records of Spanish‐speaking midwifery clients at Mary's Center between July 1, 2021, and August 31, 2023, and client satisfaction surveys for T‐GPNC. The T‐GPNC intervention was offered only to Spanish‐speaking clients in this timeframe. Our main outcome of interest was the adequacy of prenatal care, measured by generating an Adequacy of Prenatal Care Utilization Index. We conducted a thematic analysis of the surveys that included open‐ended and categorical questions.

**Results:**

The study sample for adequacy of prenatal care included 141 T‐GPNC and 193 IPC clients. The majority of clients were Latinx, and 97.9% received public insurance or were uninsured. T‐GPNC clients were significantly more likely to receive adequate or adequate‐plus care at 67.9% compared to 39.1% for IPC clients (χ^2^(2) = 45; *P* < .001). Among T‐GPNC clients who completed the satisfaction survey (n = 47; 33% response rate), they reported a high level of satisfaction (mean [SD] 4.85 out of 5 [0.63]). Qualitative themes included gaining knowledge, shared experiences, and systems‐level support.

**Discussion:**

T‐GPNC provided critical support to Spanish‐speaking clients during the COVID‐19 pandemic, producing higher rates of adequate prenatal care for T‐GPNC clients than for those receiving IPC and resulting in highly satisfied T‑GPNC clients. These statistically significant and clinically meaningful results indicate the value added by implementing T‐GPNC.

## INTRODUCTION

### Background on Telehealth, Group Prenatal Care, and COVID‐19

Telehealth group prenatal care (T‐GPNC) is an innovative care delivery method that was borne out of necessity during the COVID‐19 pandemic and incorporates the essential elements of the CenteringPregnancy group prenatal care program with telehealth.^1^ Group prenatal care is an evidence‐based model of care that has 3 core components: health care in a group space, interactive learning, and community building.^2^ Telehealth prenatal care can include synchronous audio or video visits, electronic communication, and remote client monitoring.^3^, ^4^, ^5^, ^6^, ^7^, ^8^ The use of telehealth for perinatal care increased significantly during the COVID‐19 pandemic, resulting in high levels of client satisfaction.^9^, ^10^, ^11^, ^12^ Its use reduced barriers to in‐person care, such as transportation and childcare.^10^, ^11^ The rapid expansion of telehealth led many perinatal providers to advocate for equitable access to these services via translation services, culturally congruent providers, home monitoring equipment, and digital literacy.^10^, ^11^, ^13^, ^14^

**Quick Points**

✦ Telehealth group prenatal care (T‐GPNC) provided critical support to a marginalized community during the COVID‐19 pandemic, resulting in higher rates of adequate prenatal care for T‐GPNC clients compared with individual prenatal care clients who also received telehealth.
✦ There is no prior literature on the evaluation of T‐GPNC.
✦ T‐GPNC clients were highly satisfied with their care.
✦ We recommend that midwives implement this model with technology and equipment resources that ensure equitable access to telehealth care.

There is significant literature on the positive outcomes of increased prenatal care visit attendance and client satisfaction for participants for in‐person group prenatal care.^2^ There is little to no published literature on group telehealth prenatal care and its effect on prenatal care adequacy or client satisfaction beyond the description of this innovation previously published by Daily et al.^1^ This article therefore addresses the gap in the literature for this innovative combination of group prenatal care and telehealth.

The COVID‐19 pandemic had a disproportionately negative impact on perinatal health for Latinx individuals, who were less likely to receive care and experienced higher rates of COVID‐19 infection and related morbidity and mortality than the national average.^15^, ^16^, ^17^, ^18^ The maternal mortality ratio for Latinx people increased significantly during the COVID‐19 pandemic, from 18.2 to 27.5 per 100,000 live births.^18^ Latinx birthing people were more likely to face barriers to care due to the intersection of systemic racism with social determinants of health—including reductions in transportation, access to technology, and childcare—that contribute to higher rates of COVID‐19 infection.^13^, ^16^, ^17^, ^18^

### Adequacy of Prenatal Care for the Latinx Community

Adequate prenatal care is defined as initiating prenatal care prior to 16 weeks’ gestation and attending 80% or more of the expected number of visits once enrolled.^19^, ^20^, ^21^ It can be measured using the Adequacy of Prenatal Care Utilization Index (APNCUI), which considers gestational age at the time of enrollment and the number of observed versus expected visits once enrolled, per the standard visit schedule set by the American College of Obstetricians and Gynecologists (ACOG).^19^, ^20^, ^21^ See Table 1 for a description of the APNCUI.^21^

**Table 1:** Adequacy of Prenatal Care Utilization Index

Category	Criteria
Adequate plus	Initiated by 16 weeks’ gestation and 110% or more of recommended visits received
Adequate	Initiated by 16 weeks’ gestation and 80%‐109% of recommended visits received
Intermediate	Initiated by 16 weeks’ gestation and 50%‐79% of recommended visits received
Inadequate	Initiated after 16 weeks’ gestation or less than 50% of recommended visits received

Source: Kotelchuck.^21^

The majority (69.8%) of pregnant Latinx individuals in the United States receive adequate prenatal care, slightly below the national rate of 75.6%.^22^ Over a 3‐year period (2019‐2021), the rate of inadequate prenatal care for Latinx people rose to 16.2%, whereas the national rate stabilized at 14.5%.^22^ The timing of initiation of prenatal care has a strong influence on the calculation of adequate prenatal care as defined by the APNCUI; prenatal care must begin before 16 weeks in order to achieve a designation as “adequate.”^19^, ^20^, ^21^ Latinx people are more likely to start prenatal care after the first trimester, which influences the outcome of prenatal care adequacy.^22^, ^23^ The reasons for a delay in care among those who identify as Latinx, including recent immigrants and US citizens, include language barriers, immigration status, transportation, mental health, lack of literacy in the health care system, health insurance coverage, finances, and barriers to Medicaid applications.^24^, ^25^, ^26^ Latinx people have slightly higher rates of late prenatal care initiation compared with the national rate (14.8% vs 11.1%).^22^ Late prenatal care is defined as care that starts after 16 weeks’ gestation.^22^

Pregnant people living in the District of Columbia (DC) experience lower rates of adequate prenatal care and access prenatal care later than pregnant people living in other parts of the United States.^27^ In 2023, 73% of pregnant people in DC initiated care within the first trimester, 18.4% started in the second trimester, and 8.6% initiated care in the third trimester or received no prenatal care.^27^ Prenatal care can be categorized by the APNCUI as intermediate, adequate, or adequate plus if it is initiated by 16 weeks’ gestation.^19^, ^20^, ^21^ Intermediate care indicates 50% to 79% of the recommended visits were received; adequate care indicates 80% to 109% of the recommended visits were received; and adequate plus indicates 110% of the recommended visits were received.^19^, ^20^, ^21^ Inadequate care is initiated after 16 weeks’ gestation or if less than 50% of the recommended visits were received.^19^, ^20^, ^21^ In DC in 2023, 64.8% of pregnant people received adequate or adequate‐plus care, 14.3% received intermediate care, and 21% received inadequate care.^27^ Latinx pregnant people living within DC had lower rates of early prenatal care initiation (65.9% vs 72.2%) and higher rates of late or no prenatal care (9.8% vs 8.9%) than the greater DC population.^27^ Latinx pregnant people had higher rates of inadequate prenatal care (23% vs 21.1%) in comparison with the wider population of pregnant people in DC.^27^ There are clear disparities in perinatal health outcomes that result from this inadequate prenatal care for Latinx birthing people in DC. Patient engagement in health care refers to the degree to which patients participate in recommended services.^28^ It can be influenced by their trust and relationship with their provider, access to care, and a personal commitment to wellness.^28^

DC is characterized by deep historical inequities that impact Black and, increasingly, Latinx people in the city through economic disenfranchisement, exclusionary housing policies, and underinvestment in health and social infrastructure.^29^ There are 8 federally qualified health centers (FQHCs) within DC, including Mary's Center, funded by the Health Resources and Services Administration (HRSA) that report out data via the Health Center Program Uniform Data System on health care utilization and perinatal outcomes. Mary's Center served the highest volume of prenatal clients among FQHCs in 2023, assisting 2383 of the 4811(49.5%) clients.^30^ During the COVID‐19 pandemic, the rate of early entry into prenatal care at Mary's Center decreased from 58.31% in 2021 to 48.13% in 2023.^30^ Across DC in 2023, 59.58% of pregnant clients who accessed care at these HRSA‐funded FQHCs did so in the first trimester.^30^ The objective of this project evaluation, therefore, focused on Latinx pregnant people who live in the DC area and their experience with adequate prenatal care under these pandemic conditions and with the new telehealth care modality.

## METHODS

### Setting

Mary's Center is an FQHC with 5 clinics in Maryland and DC that serves over 55,000 unique clients annually who are primarily Latinx (64%).^31^ The majority of clients have either Medicaid or other public insurance (65%) or are uninsured (24%). Mary's Center serves the highest percentage of uninsured clients in DC.^31^ Mary's Center has a comprehensive and innovative model of perinatal care provided by midwives, physician assistants, and obstetricians. Eligible clients also receive education and social services, including home visiting and connection to the Special Supplemental Nutrition Program for Women, Infants, and Children. In 2023, 92% of pregnant clients gave birth to a newborn with a healthy birth weight (over 5.5 lbs), 48% entered prenatal care in the first trimester, and 90% of home‐visiting participants attended their postpartum care appointments.^31^ Mary's Center midwives have been offering CenteringPregnancy (group prenatal care) in person since 2012 and individual telehealth perinatal visits since 2019.^1^ This background and experience provided the foundation for the creation of the T‐GPNC program in 2020.^1^ The purpose of this clinical innovation was to reach Latinx clients during the COVID‐19 pandemic with safe prenatal care, education, and community support.^1^

### Intervention

The intervention of T‐GPNC is defined as group prenatal care offered virtually that includes an individual telehealth clinical assessment, group education and support, along with essential in‐person individual care visits.^1^ T‐GPNC at Mary's Center included a minimum of 5 in‐person individual prenatal care (IPC) visits at a set interval to ensure essential assessments and interventions (ie, physical examination, laboratory studies, vaccines, ultrasounds, and medications) could be completed. Full details of the development and implementation of the innovative T‐GPNC and description of group sessions were previously described.^1^ IPC is standard prenatal care that includes both in‐person and telehealth visits per the COVID‐19 pandemic protocols. IPC had a minimum of 5 in‐person visits with 5 to 7 telehealth visits interspersed. All clients received telehealth during this timeframe due to COVID‐19 pandemic protocols, via T‐GPNC or IPC visits. Mary's Center clients met for a prenatal enrollment visit at the start of their pregnancy, and if they were identified as healthy, low risk, Spanish speaking, and eligible for midwifery care, they were routinely offered the choice of T‐GPNC or IPC. This self‐selection continued in early pregnancy; individuals could select T‐GPNC up until the second group session, held between 18 to 22 weeks’ gestation. The T‐GPNC intervention was only offered in Spanish in this timeframe because there were not enough English‐speaking clients who were both eligible and interested in T‐GPNC. See Table 2 for a description of the IPC and T‐GPNC visit schedule at Mary's Center.

**Table 2:** Mary's Center Prenatal Visit Schedule: Individual Prenatal Care and Telehealth‐Group Prenatal Care

Gestational age, wk	IPC	T‐GPNC	Essential Care Measures
6‐10	Telehealth individual visit for IPC and T‐GPNC clients		Prenatal enrollment
10‐14	In person	In‐person, individual	NOB Physical examination and laboratory tests Vitals Viability and dating ultrasound^a^ Cervical cancer screening Genetic screening Vaccines (flu and COVID‐19)
14‐18	Telehealth	Telehealth group session #1	Telehealth assessment^b^
18‐22		Telehealth group session #2	Telehealth assessment^b^
20	In person	In‐person, individual	ROB Labs, vitals, fetal heart tones Anatomy ultrasound^a^
22‐26	Telehealth	Telehealth group session #3	Telehealth assessment^b^
28	In person	In‐person, individual	ROB Third‐trimester labs (CBC, GDM, STI), vitals, fetal heart tones Tdap vaccine Rhogam as needed
26‐30		Telehealth group session #4	Telehealth assessment^b^
30	Telehealth		Telehealth assessment^b^
30‐34		Telehealth group session #5	Telehealth assessment^b^
32	In person	In‐person, individual	ROB Vitals, fetal heart tones Fetal growth Fetal position
34	Telehealth		Telehealth assessment^b^
34‐36		Telehealth group session #6	Telehealth assessment^b^
36‐38		Telehealth group session #7	Telehealth assessment^b^
36	In person	In‐person, individual	ROB Vitals, fetal heart tones GBS, STI Fetal position
37	Telehealth		Telehealth assessment^b^
38	Telehealth		Telehealth assessment^b^
38‐40		Telehealth group session #8	Telehealth assessment^b^
39	In person	In‐person, individual	ROB Vitals, fetal heart tones Birth planning
40	Telehealth	In‐person, individual	Telehealth assessment^b^

Abbreviations: CBC, complete blood count; GA, gestational age; GBS, group B streptococcus vaginal‐rectal swab; GDM, gestational diabetes mellitus; IPC, individual prenatal care; NOB, initial prenatal care visit; ROB, return prenatal care visit; STI, sexually transmitted infection; Tdap, tetanus, diphtheria, and pertussis vaccination; T‐GPNC, telehealth group prenatal care.

a Ultrasound performed at Mary's Center ultrasound clinic located at Ontario Rd DC site or partner sites as separate visit.

b Telehealth assessment included prenatal visit, blood pressure, weight, heart rate, pregnancy symptoms, fetal kick counts (as appropriate per GA).

### Theoretical Framework

**Figure 1:**
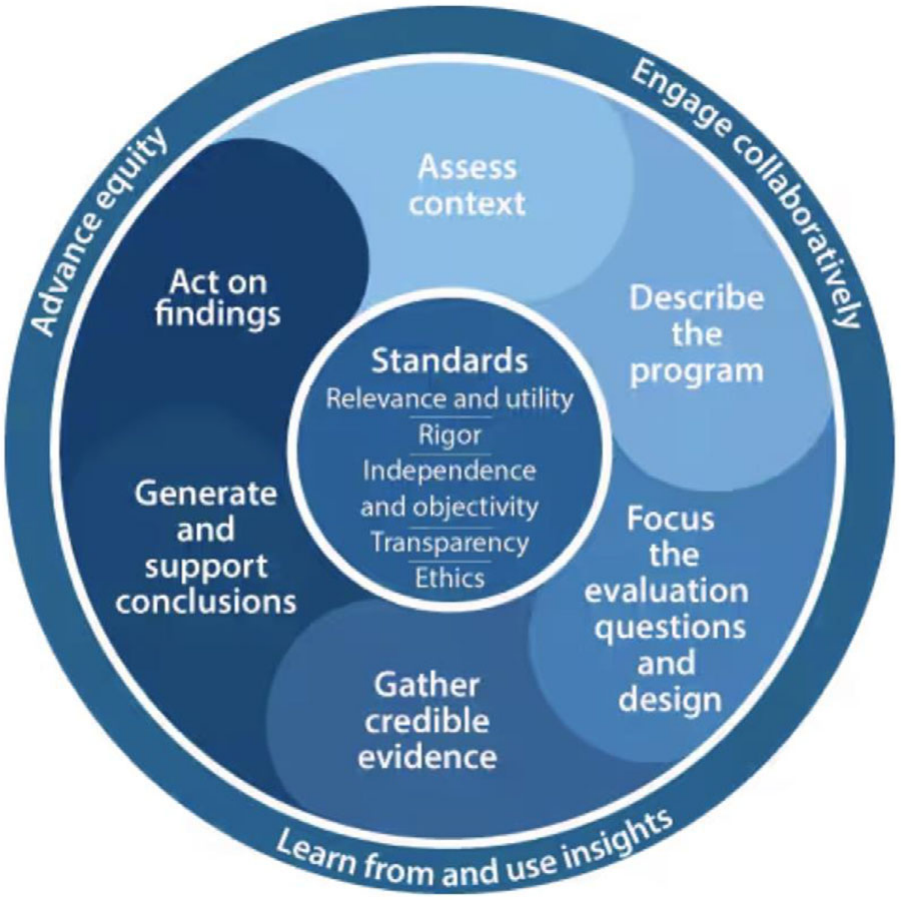
Centers for Disease Control and Prevention Framework for Program Evaluation in Public Health Source: CDC.^32^

We used the Centers for Disease Control and Prevention Framework for Program Evaluation in Public Health as the theoretical framework for this program evaluation.^32^ The framework includes 3 cross‐cutting actions, 5 evaluation standards, and 6 evaluation steps (Figure 1).^32^ We engaged collaboratively with staff, clinicians, and administrators at Mary's Center by assessing the current context of perinatal care at Mary's Center, establishing the program evaluation aims, selecting appropriate outcome measures, undergoing a formal review by the Mary's Center Research Review Committee (MCRRC), and extracting electronic health record (EHR) data to achieve program evaluation aims. The study team and Mary's Center collaborators clarified a joint commitment to advancing health equity for all birthing people by evaluating innovative models of care that may increase accessibility and acceptability. All study findings were discussed and reviewed with the Mary's Center team at each stage of the program evaluation, including data cleaning, data analysis, and dissemination of findings (including co‐authorship on manuscripts and presentations) to ensure joint ownership, accountability, and power‐sharing. Mary's Center promotes a model of social change that recognizes individuals and families need a combination of health care, social services, and education to achieve wellness, stability, and economic independence.^31^ Mary's Center and our research team are committed to learning from and using the insights generated from this program evaluation to develop the evidence in support of innovative models of prenatal care that maintain or expand access for people for whom in‐person care is untenable. The Strengthening the Reporting of Observational Studies in Epidemiology (STROBE) checklist was used as a guideline to report the study findings (Supporting Information: Appendix S1).

### Evaluation Design

We conducted a convergent mixed‐methods program evaluation to compare the adequacy of prenatal care for T‐GPNC clients with those receiving IPC, and we explored client satisfaction with T‐GPNC. We analyzed the quantitative and qualitative data and then integrated the results. We compared the adequacy of prenatal care for pregnant people who received T‐GPNC to those who received IPC. The Mary's Center outcomes team extracted the jointly selected data points from the EHRs and conducted quality checks in partnership with the first and last author. The CenteringPregnancy Coordinator at Mary's Center provided the client satisfaction survey results data, which were then translated from Spanish by the main author. The use of client satisfaction surveys ensured client participation in this time‐ and resource‐limited evaluation.

### Positionality Statement

Catherine “Katie” Daily is a bilingual (English/Spanish), White, cisgender, heterosexual, mother trained as a nurse‐midwife who practices at the project site, Mary's Center. Drs. Marea, Gresh, and Crowell were mentors on Dr. Daily's DNP project, upon which this manuscript was based. Ashley Gresh is a White, cisgender, heterosexual, nondisabled woman and mother trained as a nurse‐midwife and public health nurse. Elizabeth R. Hamilton is a Black, cisgender, heterosexual woman who is a mother and a first‐generation American born to Jamaican immigrants. Elizabeth served in health care quality leadership roles in community health settings, primarily federally qualified health centers, and is the chief quality officer for the San Francisco Department of Health. Nancy Crowell is a White, cisgender female. Germán “Gustavo” Alva Martinez is a bilingual Latino, cisgender, heterosexual data analyst originally from Peru with a degree in statistics. Christina X. Marea is a bilingual (English/Spanish) Latina, cisgender, heterosexual, neurodiverse parent who has given birth twice and practices as a nurse‐midwife at a federally qualified health center in Washington DC.

#### Ethics

This study was approved by the MCRRC, which includes members from senior leadership, outcomes, social services, and health care providers. This study was approved via expedited review by the Georgetown University Medical Center Institutional Review Board on September 6, 2023 (IRB Study #00006633).

### Sample

**Figure 2:**
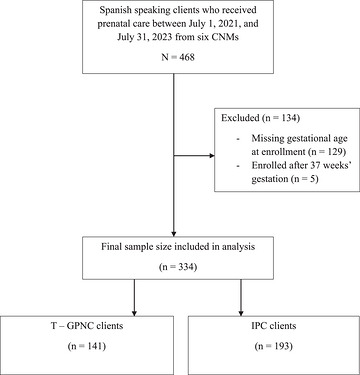
Client Flowchart Abbreviations: CNM, certified nurse‐midwife; IPC, individual prenatal care; T‐GPNC, telehealth group prenatal care.

The sample for this program evaluation included Mary's Center clients who received prenatal care from midwives during the COVID‐19 pandemic from the onset of regular T‐GPNC session offerings (July 1, 2021) until they resumed in‐person GPNC (August 31, 2023). Eligibility criteria further included only clients who received care from the 6 midwives who provided both T‐GPNC sessions and IPC and who identified Spanish as their primary language. Clients who enrolled after 37 weeks’ gestation or whose gestational age was unable to be determined using existing EHR data (no documented estimated due date, no date document at new obstetrician appointment, or both missing) were excluded from the data. See Figure 2 for the client flowchart.

### Measures

#### Demographic Data

We extracted demographic data for eligible clients from the EHR, including age, parity, zip code, insurance type, language, race, and ethnicity.

#### Health Care Engagement

The measures of health care engagement included gestational age at time of entry into prenatal care, total number of prenatal care visits (by visit type), gestational age at time of birth, number of no‐show visits (by visit type), and documented blood pressure. We considered the number of no‐shows (IPC vs T‐GPNC) as a proxy measure to capture barriers to care by modality of visit.

#### Adequacy of Prenatal Care

We measured the adequacy of prenatal care using 2 measures. First, we extracted from the EHR whether there was documented blood pressure for each visit, a clinically important vital sign that should be documented at each prenatal visit.^19^ Second, we determined the APNCUI. This is a valid and reliable measure that aligns with the clinical practice guidelines set by ACOG for prenatal care for low‐risk pregnancies.^19^, ^20^, ^21^ The APNCUI considers the timing of initiation of prenatal care and the number of observed versus expected visits once enrolled, according to the ACOG standard visit schedule.^19^, ^20^, ^21^ The APNCUI requires 3 client data points: the gestational age at time of entry into prenatal care, the total number of prenatal care visits, and the gestational age at the time of birth. Adequacy is then calculated based on whether the client enrolled in prenatal care by 16 weeks and received the expected number of visits. Adequate care is then defined as initiation of prenatal care by 16 weeks’ gestation and 80% or more attendance at routine visits. Prenatal care is classified as inadequate, intermediate, adequate, or adequate plus, according to the APNCUI scoring guidelines.^21^ See Table 1 for a description of the APNCUI.^21^

#### Client Satisfaction with T‐GPNC

The CenteringPregnancy program requires that all approved sites implement a client satisfaction survey provided in the bilingual Centering Healthcare Institute materials. Mary's Center formatted the surveys into a Google form and sent the link via text message to clients at the final group session. Clients could complete the anonymous survey on their mobile devices. The survey includes categorical questions on topics in which clients were asked to classify each topic as *discussed/not discussed*, and Likert‐type ordinal responses to rate the content as *very useful*, *useful*, or *not useful*. These topics included normal changes in pregnancy, nutrition, exercises and relaxation, pregnancy complications, breastfeeding and infant feeding, sexuality and family planning, family and relationships, interpersonal violence, labor and birth, newborn care, postpartum care, and emotional changes and depression. Clients then indicated if they liked receiving T‐GPNC (*yes/no*) and rated their overall satisfaction with T‐GPNC on a Likert scale of 1 to 5, with 1 being described as the worst and 5 as the best. The survey also included 2 open‐ended questions: (1) What would you say to other women about receiving prenatal care in this way? and (2) How could we improve your prenatal care experience?

### Analysis

We used SPSS Statistics Version 29.0 for data analysis. Descriptive statistics were used to describe the sample and the variables of interest. Frequency distributions (n and percent) were used for nominal and ordinal variables; means and standard deviations were used for interval/ratio variables. Comparisons between groups used *t* tests to compare means of interval/ratio variables (such as gestational age at enrollment or number of visits completed) and chi square for nominal/ordinal variables (such as state of residence and adequacy of care rating). An experienced statistician contracted by Georgetown University completed the data analysis.

#### Client Satisfaction Survey

The quantitative results from the client satisfaction surveys were analyzed using descriptive statistics. The open‐ended questions were analyzed by an inductive thematic analysis.^33^ To begin the process, the first author, a bilingual midwife, translated the open‐ended survey results into English. The translated responses were then coded independently by 2 members of the study team (KD and AG). We followed the 6 phases of thematic analysis: (1) familiarizing yourself with your data, (2) generating initial codes, (3) searching for themes, (4) reviewing themes, (5) defining and naming themes, and (6) producing the report. To increase the rigor, reliability, and trustworthiness of the data, we used a checklist developed to promote adherence to the phases of thematic analysis.^33^ The coders then met to arrive at a consensus on emerging themes.^34^

## RESULTS

### Demographics

A total of 334 clients were included in the analysis, with 141 (42.2%) T‐GPNC clients and 193 (57.8%) IPC clients (Table 3). A total of 134 clients were excluded from the initial sample (Figure 2). T‐GPNC clients were significantly more likely to be from DC (51.8% vs 47.5%), and IPC clients were more likely to be from Maryland (66.8% vs 33.2%; χ^2^(2), 13.8; *P* = .001). There were no statistically significant differences in client demographics, including race, insurance type, and age. People in the T‐GPNC group enrolled at a significantly earlier gestational age (15.4 weeks) than people in the control group (15.4 vs 18.8 weeks; *t*(18.8) = 5.7; *P* < .001). The sample included one hybrid T‐GPNC cohort of 9 clients that experienced 3 in‐person GPNC sessions and 5 T‐GPNC sessions.

**Table 3:** Client Demographics by Group

Demographics	T‐GPNC	IPC	*P* Value
**State, n (%)**			.001
Washington, District of Columbia	73 (51.8)	64 (33.2)	
Maryland	67 (47.5)	129 (66.8)	
Virginia	1 (0.7)	0 (0)	
**Race, n (%)**			.054
Hispanic or Latinx	121 (99.2)	172 (98.3)	
Multirace	17 (28.3)	46 (45.1)	
Other	1 (1.7)	4 (3.9)	
White	42 (70.0)	52 (51.0)	
**Insurance Type, n (%)**			.245
Private	3 (2.1)	61 (31.6)	
Public	81 (57.4)	128 (66.3)	
Uninsured	57 (40.4)	61 (31.6)	
**Maternal age, mean (SD), y**	28.7 (5.9)	29.5 (6.7)	.228
**Gestational age at enrollment, mean (SD), wk**	15.4 (4.1)	18.8 (6.9)	<.001

Abbreviations: IPC, individual prenatal care; T‐GPNC, telehealth group prenatal care.

### Adequacy of Prenatal Care

#### Adequacy of Prenatal Care Utilization Index

The T‐GPNC group completed significantly more prenatal visits than the IPC group (15.6 vs 10.3; *t*(332) = 11.3; *P* < .001). There were no differences between groups in percentages of no‐show visits. The T‐GPNC group was significantly more likely to have adequate‐plus care (62.9%) than the IPC group (62.9% vs 26.6%; χ^2^(2), 45; *P* < .001). Overall, the T‐GPNC group had significantly higher rates of adequate care (adequate and adequate plus) than the IPC group (67.9% vs 39.1%; χ^2^(2), 45; *P* < .001). The IPC group was significantly more likely to receive inadequate care than the T‐GPNC group (55.2% vs 30.7%; χ^2^(2), 45; *P* < .001).

#### Blood Pressure Documentation

The T‐GPNC group had a significantly higher percentage of individual telehealth visits with a recorded blood pressure (BP) reading compared with the IPC group (42.7% vs 13.6%; *t*(153.8) = 4.2; *P* < .001; adjusted degrees of freedom to account for unequal variances). See Table 4 for adequacy of care measures by group.

**Table 4:** Adequacy of Care Measures by Group

	T‐GPNC	IPC	*P* Value^a^
**Individual telehealth visits with BP, mean (SD), %**	42.7 (70.2)	13.6 (31.0)	<.001
**Group telehealth visits with BP, mean (SD), %**	42.0 (38.6)	NA	NA
**Recommended visits completed, mean (SD), %**	153.1 (42.0)	112.9 (37.7)	<.001
**Adequacy Ranking**			<.001
Adequate plus, n (%)	88 (62.9)	51 (26.6)	
Adequate, n (%)	7 (5.0)	24 (12.5)	
Intermediate, n (%)	2 (1.4)	11 (5.7)	
Inadequate, n (%)	43 (30.7)	106 (55.2)	

Abbreviations: BP, blood pressure; IPC, individual prenatal care; NA, not available; T‐GPNC, telehealth group prenatal care.

a *P* values based on independent samples *t* test for percent of individual telehealth visits with BP and percent of recommended visits completed; *P* values based on χ^2^ for adequacy.

### Client Satisfaction with T‐GPNC

The second aim of this program evaluation was to describe client satisfaction with T‐GPNC. Client satisfaction surveys were collected from 47 out of 141 individuals during their final T‐GPNC session (33% response rate). These surveys were collected as part of the standards for GPNC at the project site. Satisfaction surveys were not collected for IPC due to the time constraints and resource limitations for this project.

Topic usefulness ratings ranged from 2.84 to 2.98 on a 3‐point scale (1 = not very useful, 2 = somewhat useful, and 3 = very useful). Forty‐five participants (95.7%) agreed that they liked to receive prenatal care in group sessions; only 1 (2.1%) disagreed, and 1 (2.1%) did not answer. When asked to rate group care on a scale of 1 (worst) to 5 (best), the average rating was 4.85 (SD = 0.63). One person (2.1%) rated it as 1; 3 (6.4%) rated it as 4; and the remaining 43 (91.5%) rated it as 5.

The survey ended with 2 open‐ended questions that all survey respondents completed regarding what they would tell others about T‐GPNC and how it could be improved. Three themes emerged: (1) individuals gaining knowledge, (2) interpersonal shared experiences, and (3) systems‐level support. Client quotes were used to illustrate these themes. One client reported learning about new health topics: “It is a great way to receive care and information about many topics that we did not know about” (Client 1). Another client described feeling in community with others through interpersonal shared experiences: “It is an interactive way to share experiences, emotions during pregnancy with other moms, and feel like during this pregnancy journey you are also in community” (Client 2). Clients also reported feeling supported at a systems level, as one client said, “They help and offer good support and teach us many things that we did not know about” (Client 3).

All clients stated T‐GPNC was a positive experience; for example, “It is a very fun and interesting and educational program for pregnant women” (Client 4). Most stated that no improvements were needed; for example, “It is the best care. You do not need to improve it” (Client 5). Many clients took this opportunity to express gratitude for the experience.

## DISCUSSION

Disruptions to the accessibility of in‐person prenatal care are increasing. Infectious disease outbreaks (eg, COVID‐19), extreme weather events, and sociopolitical disruptions (eg, targeted “immigration” enforcement at health care facilities) can make attending in‐person care difficult, and sometimes dangerous, for pregnant people.^35^ It is imperative that perinatal care providers are prepared to nimbly respond to these disruptions with flexible and innovative approaches to ensure continuity of care and positive outcomes for people made vulnerable by systematic and structural failures to ensure health equity at a societal level.^36^

The COVID‐19 pandemic had an inequitable impact on Hispanic/Latinx communities, who were deeply impacted by structural barriers to care, including reduced transportation and childcare.^13^, ^16^, ^17^, ^18^ Despite these barriers, Hispanic T‐GPNC clients were more likely to stay engaged in prenatal care compared with those enrolled in individual care, providing evidence to support the use of T‐GPNC to provide adequate prenatal care, particularly when access may be limited due to emergencies. The high rate of client satisfaction with T‐GPNC supports its use as an acceptable care modality, particularly as it addresses the priorities of Latinx clients for social support, eases logistical burdens, and promotes cultural awareness.^26^ Those enrolled in T‐GPNC had the benefit of the CenteringPregnancy program coordinator and continuity of the midwife, resulting in trusting relationships, further augmenting the social support provided by the group care model.^37^, ^38^, ^39^, ^40^ The IPC clients did not have this guaranteed continuity of care, although it was not measured and may have occurred. Spanish language providers, effective education, and access to pregnancy prevention are effective promoters of early entry into prenatal care for Latinx pregnant people, which were all present in our T‐GPNC program.^23^, ^24^, ^26^ T‐GPNC clients received support from fellow clients, facilitators, and connections to community resources—known benefits of GPNC.^39^, ^40^, ^41^

The T‐GPNC clients were significantly more likely to enroll in prenatal care at an earlier gestational age than IPC clients; however, both groups enrolled in care on average after 16 weeks’ gestation, the Mary's Center Health Employer Data Information Set–aligned quality measure of first‐trimester entry to prenatal care.^42^ The study findings of late enrollment in prenatal care align with both the national and DC rates for Latinx pregnant people.^22^, ^27^ Pregnant people who enroll in prenatal care early are more likely to receive adequate prenatal care, at least partially because early prenatal care is one component of the measure.^43^ First‐trimester entry to care as a measure of adequacy and quality is challenging, given the structural barriers to care faced by many Latinx people, the FQHC‐mandated policy allowing enrollment at any gestational age, and for facilities caring for primarily uninsured and publicly insured people.^43^, ^44^, ^45^ Medicaid expansion, including access for immigrants, may lead to greater equity in adequate prenatal care between individuals who identify Spanish as their primary language and those who identify English as their primary language.^23^, ^25^ The inclusion of early enrollment as a measure of care adequacy in a population that enrolls late due to structural barriers can penalize FQHCs that provide excellent prenatal care and important avenues to access with mandated sliding scales. Because clients self‐selected into T‐GPNC, and were able to do so until 18 to 22 weeks’ gestation, this may have led to selection bias, leading to higher rates of adequacy for T‐GPNC clients.

The documentation of BP readings was lower than expected for both T‐GPNC and IPC clients. Based on the first author's clinical experience providing T‐GPNC, we suspect that BPs were collected by clients at home but were inconsistently documented in the EHR during the telehealth visits. None of the BP monitors distributed automatically imported BPs into the EHR; instead, they had to be read to staff and then transcribed, increasing the opportunities for human error. Other COVID‐era studies found that fewer than half of those receiving telehealth prenatal care had an automatic BP cuff, and around one‐third used it; conversely, there were high levels of comfort among pregnant people with self‐monitoring abilities.^11^, ^12^, ^46^, ^47^ Yet T‐GPNC clients had a much higher rate of a documented BP reading than IPC clients, which may be due to increased patient education in T‐GPNC and more consistent distribution of equipment facilitated by grant funding.

Telehealth increases access to care across populations; however, lower‐income clients and Latinx individuals may self‐select in‐person care due to their limitations in accessing high‐quality devices, reliable Wi‐Fi, and digital literacy.^9^, ^47^, ^48^ Prior research indicates that a key facilitator of telehealth use among lower‐income clients was education on how to use technology and home monitoring equipment.^49^ The T‐GPNC program provided personalized training on the use of the Zoom platform and the equipment by the program coordinator, and they reinforced this in group sessions.

There are screening tools available for providers to assess clients for levels of digital literacy and access to the internet and phone, which can be implemented at care visits.^50^ Those who identified barriers could be offered digital literacy training, either through resources identified at the clinic or by leveraging community resources such as the public library system.^9^, ^12^, ^51^ It would be beneficial to implement an interactive platform for all telehealth clients via digital BP monitors that upload client results directly into a portal accessible by providers.^52^ Other telehealth perinatal care programs incorporated the use of handheld Dopplers, adding self‐measured fundal height into their care with success.^9^, ^12^ We need to implement measures to ensure continuous access to prenatal care in the face of the societal disruptions from infectious disease outbreaks, environmental disasters, and sociopolitical events.

ACOG recommends increasing the use of telehealth for average‐risk pregnant people.^3^ The literature on the use of telehealth for routine prenatal care indicates that telehealth is not inferior to in‐person care, with no increased risk for adverse maternal or neonatal outcomes.^9^, ^45^, ^51^, ^53^, ^54^, ^55^ The results from this program evaluation further support these findings. In order to ensure that telehealth, group, and combined telehealth group models of care are sustainable, there must be adequate reimbursement that includes support for home supplies (eg, BP monitors) and appropriate support staff to manage scheduling, technological support, and care coordination when in‐person follow‐up is required. Although telehealth payment models vary widely by state, reimbursement strategies should move beyond fee‐for‐service models to include value‐based payment and cost‐effectiveness approaches, particularly in light of growing barriers to access for marginalized populations and those impacted by maternity service closures.

The majority of clients indicated that they were satisfied with their T‐GPNC experience and that most topics were useful. Client satisfaction is overall high for in‐person GPNC, and our results confirm that holds true for T‐GPNC as well.^56^ Telehealth prenatal care also has high levels of client satisfaction.^45^, ^51^

### Strengths and Limitations

Our study included a robust sample size, leading to statistically significant and clinically meaningful results and the inclusion of client perspectives via satisfaction surveys. There were multiple limitations in the EHR data set that may have resulted from documentation errors or challenges in the data‐capture process rather than insufficient clinical care, which need to be explored. We were only able to assess for adequacy of care up to 37 weeks’ gestation due to a lack of consistent documentation of gestational age at birth. The subset of 20 clients who indicated interest in T‐GPNC but were unable to participate in any group care visits may represent a missed opportunity to assess and address barriers to telehealth. There may have been selection bias as clients self‐selected T‐GPNC or IPC, and those who chose T‐GPNC may have already been more engaged in their care. We present here a retrospective program evaluation of T‐GPNC during a pandemic, which may have impacted data quality. There was a high proportion of patients with a missing estimated date of birth (EDB), which may be due to patients switching to outside providers before establishing an EDB, unsure dating and inability to access confirmation sonography, or late entry with birth before EDB was established. Future randomized controlled trials should be explored to assess the adequacy of individual versus group telehealth prenatal care, include more multidimensional measures of adequacy, and ensure more robust data collection and data quality.

There were multiple limitations in evaluating client satisfaction. Only 33% of clients who participated in T‐GPNC completed surveys. There may be a positive bias, as those unsatisfied with T‐GPNC could have transferred into IPC prior to the implementation of surveys, and the surveys were optional, without an incentive offered for survey completion. The surveys were not linked to individual clients, so we cannot correlate results to client engagement. No equivalent surveys were collected for IPC clients to provide a comparison of client experiences. This was a retrospective evaluation, and there was no existing process to collect client satisfaction data regarding IPC.

Future research should prospectively explore maternal and infant outcomes, including potential randomized controlled trials that compare T‐GPNC to other prenatal care modalities. T‐GPNC can be adapted for use with other linguistic and cultural groups, as well as those in rural communities.

### Practice Implications

The findings from this evaluation indicate that T‐GPNC is a valuable program to increase the adequacy of prenatal care and ensure continuous access to care during disruptions. It follows the guidelines released to integrate appropriate, tailored telehealth visits into routine prenatal care.^3^, ^57^, ^58^ It disrupts the standards set decades ago for frequency and modality of prenatal care visits, which were based on limited evidence.^19^, ^57^, ^58^ The T‐GPNC program follows standards of telehealth excellence with the inclusion of team members across disciplines, shared community resources, and the social connections between clients.^1^, ^59^

The program addressed issues of equitable access to telehealth by training clients how to access this technology.^1^, ^11^, ^51^ T‐GPNC can be implemented at other FQHCs and community health centers with resources in place for digital literacy training and care coordination. Factors to consider include creating cohorts of preferred language and engaging culturally congruent community health workers. Programs could consider expanding T‐GPNC to include more online social connections through digital platforms. Unfortunately, Mary's Center is no longer implementing the T‐GPNC program at this time due to a lack of funding for a program coordinator and supplies.

## CONCLUSION

The T‐GPNC model is an innovative care method developed in response to the COVID‐19 pandemic, resulting in adequate prenatal care for the majority of clients and high levels of client satisfaction. This program evaluation fills a gap in the literature on the implementation of T‐GPNC. It presents T‐GPNC as equivalent to IPC in terms of adequacy, and in most measures, provided more than adequate care. The use of T‐GPNC is an important intervention to offer structurally marginalized pregnant people to start addressing the rising inequities of perinatal health outcomes in the United States.

## CONFLICT OF INTEREST

Research reported in this publication was supported by the National Center for Advancing Translational Sciences of the National Institutes of Health under Award Number KL2TR001432. The authors have no conflicts of interest to disclose.

## Supporting information




**Appendix S1**. Strengthening the Reporting of Observational Studies in Epidemiology (STROBE) Checklist

## References

[1] Daily C , Gresh A , Hamilton ER , Marea CX . Adapting group prenatal care for telehealth: A COVID‐era innovation to address barriers to care for Latinx clients. J Midwifery Womens Health. 2024;69(6):945‐951. doi:10.1111/jmwh.13701 39463123 PMC11648179

[2] Rising SS , Quimby CH . The CenteringPregnancy Model: The Power of Group HealthCare. Spring Publishing Company; 2016.

[3] The American College of Obstetricians and Gynecologists. Implementing telehealth in practice. Obstet Gynecol. 2020;135(2):e73‐e79. doi:10.1097/AOG.0000000000003671 31977796

[4] Avercenc L , Ngueyon Sime W , Bertholdt C , et al. Improving prenatal care during lockdown: comparing telehealth and in‐person care for low‐risk pregnant women in the PROTECT pilot study. J Gynecol Obstet Hum Reprod. 2022;51(9):102445. doi:10.1016/j.jogoh.2022.102445 35882366 PMC9364754

[5] Balk EM , Konnyu KJ , Cao W , et al. Schedule of visits and televisits for routine antenatal care: A systematic review. Report No. 22‐EHC031. United States Agency for Healthcare Research and Quality; 2022. doi:10.23970/AHRQEPCCER257. 35862565

[6] Center for Connected Health Policy . State Telehealth Laws and Reimbursement Policies Report, Fall 2024. CCHP website. November 2024. Accessed November 16, 2024. https://www.cchpca.org/resources/state‐telehealth‐laws‐and‐reimbursement‐policies‐report‐fall‐2024/

[7] Weigel G , Frederiksen B , Ranji U . Telemedicine and Pregnancy Care. Kaiser Family Foundation website. February 26, 2020. Accessed April 13, 2024. https://www.kff.org/womens‐health‐policy/issue‐brief/telemedicine‐and‐pregnancy‐care/

[8] Butler Tobah YS, LeBlanc A , Branda ME , et al. Randomized comparison of a reduced‐visit prenatal care model enhanced with remote monitoring. Am J Obstet Gynecol. 2019;221(6):638.e1‐638.e8. doi:10.1016/j.ajog.2019.06.034 31228414

[9] Atkinson J , Hastie R , Walker S , Lindquist A , Tong S . Telehealth in antenatal care: recent insights and advances. BMC Med. 2023;21(1):332. doi:10.1186/s12916-023-03042-y 37649028 PMC10470141

[10] Kissler K , Thumm EB , Smith DC , et al. Perinatal telehealth: meeting patients where they are. J Midwifery Womens Health. 2024;69(1):9‐16. doi:10.1111/jmwh.13560 37641584 PMC10873126

[11] Morgan A , Goodman D , Vinagolu‐Baur J , Cass I . Prenatal telemedicine during COVID‐19: patterns of use and barriers to access. JAMIA Open. 2022;5(1):ooab116. doi:10.1093/jamiaopen/ooab116 35146379 PMC8822407

[12] Wu KK , Phillippi J , Mueller M , Lopez C , Nichols M . Telemedicine for routine prenatal care: use and satisfaction during the COVID‐19 pandemic. J Midwifery Womens Health. 2024;69(4):469‐478. doi:10.1111/jmwh.13621 38477390

[13] Britt AJ , Carlson NS , Joseph NT , Amore AD . The convergence of COVID‐19 and systemic racism: An evaluation of current evidence, health system changes, and solutions grounded in reproductive justice. J Midwifery Womens Health. 2021;66(3):298‐303. doi:10.1111/jmwh.13250 34114324 PMC9014468

[14] Smith DC , Thumm EB , Anderson J , et al. Sudden shift to telehealth in COVID‐19: A retrospective cohort study of disparities in use of telehealth for prenatal care in a large midwifery service. J Midwifery Womens Health. 2024;69(4):522‐530. doi:10.1111/jmwh.13601 38111228 PMC11182882

[15] Basile Ibrahim B , Kennedy HP , Combellick J . Experiences of quality perinatal care during the US COVID‐19 pandemic. J Midwifery Womens Health. 2021;66(5):579‐588. doi:10.1111/jmwh.13269 34432368 PMC8661618

[16] Salgado de Snyder VN , McDaniel M , Padilla AM , Parra‐Medina D . Impact of COVID‐19 on Latinos: A social determinants of health model and scoping review of the literature. Hisp J Behav Sci. 2021;43(3):174‐203. doi:10.1177/07399863211041214

[17] Poulson M , Neufeld M , Geary A , et al. Intersectional disparities among Hispanic groups in COVID‐19 outcomes. J Immigr Minor Health. 2021;23(1):4‐10. doi:10.1007/s10903-020-01111-5 33090300 PMC7579850

[18] U .S. Government Accountability Office . Maternal Health: Outcomes Worsened and Disparities Persisted During the Pandemic. GAO‐23‐105871. GAO website. October 19, 2022. Accessed November 15, 2024. https://www.gao.gov/products/gao‐23‐105871

[19] AAP Committee on Fetus and Newborn; ACOG Committee on Obstetric Practice ; Kilpatrick SJ , Papile L‐A , eds. Guidelines for Perinatal Care. 8th ed. American Academy of Pediatrics and The American College of Obstetricians and Gynecologists; 2017.

[20] Rowe S , Karkhaneh Z , MacDonald I , et al. Systematic review of the measurement properties of indices of prenatal care utilization. BMC Pregnancy Childbirth. 2020;20(1):171. doi:10.1186/s12884-020-2822-5 32183724 PMC7079477

[21] Kotelchuck M . An evaluation of the Kessner adequacy of prenatal care index and a proposed adequacy of prenatal care utilization index. Am J Public Health. 1994;84(9):1414‐1420. doi:10.2105/AJPH.84.9.1414 8092364 PMC1615177

[22] Martin JA , Osterman MJK . Changes in prenatal care utilization: United States, 2019–2021. Natl Vital Stat Rep. 2023;72(4):1‐14. https://stacks.cdc.gov/view/cdc/125706 37252688

[23] Choi S , McElfish PA , Brown CC . Disparities in prenatal care utilization among racial/ethnic and nativity subgroups in the United States. Prev Med. 2025;192:108238. doi:10.1016/j.ypmed.2025.108238 39889834 PMC11845284

[24] Camargo JT , Barral RL , Kerling EH , et al. Prenatal care utilization challenges and facilitators for a growing Latino community in the midwest. Matern Child Health J. 2023;27(10):1811‐1822. doi:10.1007/s10995-023-03733-1 37369811 PMC11251489

[25] Interrante JD , Pando C , Fritz AH , Kozhimannil KB . Perinatal care among Hispanic birthing people: differences by primary language and state policy environment. Health Serv Res. 2024;59(5):e14339. doi:10.1111/1475-6773.14339 38881220 PMC11366965

[26] Fryer K , Lewis G , Munoz C , Stuebe AM . Identifying barriers and facilitators to prenatal care for Spanish‐speaking women. N C Med J. 2021;82(1):7‐13. doi:10.18043/ncm.82.1.7 33397748 PMC7927271

[27] March of Dimes: Peristats. Prenatal Care . March of Dimes website. Updated January 2024. Accessed March 14, 2025. https://www.marchofdimes.org/peristats/data?reg=99&top=5&stop=29&lev=1&slev=4&obj=1&sreg=11&creg

[28] Higgins T , Larson E , Schnall R . Unraveling the meaning of patient engagement: A concept analysis. Patient Educ Couns. 2017;100(1):30‐36. doi:10.1016/j.pec.2016.09.002 27665500

[29] King CJ , Buckley BO , Maheshwari R , Griffith DM . Race, place, and structural racism: A review of health and history in Washington, D.C. Health Aff (Millwood). 2022;41(2):273‐280. doi:10.1377/hlthaff.2021.01805 35130070

[30] District of Columbia Health Center Program Uniform Data System (UDS) Data. HRSA website. 2024. Accessed December 12, 2024. https://data.hrsa.gov/tools/data‐reporting/program‐data/state/DC

[31] 2024–2025 Annual Report. Mary's Center website. Accessed November 8, 2024. https://www.maryscenter.org/about‐us/annual‐report/highlights/

[32] Centers for Disease Control and Prevention . Framework for program evaluation in public health. MMWR Recomm Rep. 1999;48(RR‐11):1‐40. https://www.cdc.gov/mmwr/PDF/rr/rr4811.pdf 10499397

[33] Ahmed SK , Mohammed RA , Nashwan AJ , et al. Using thematic analysis in qualitative research. J Med Surg Public Health. 2025;6:100198. doi:10.1016/j.glmedi.2025.100198

[34] Hsieh HF , Shannon SE . Three approaches to qualitative content analysis. Qual Health Res. 2005;15(9):1277‐1288. doi:10.1177/1049732305276687 16204405

[35] Zhong L , Lopez D , Pei S , Gao J . Healthcare system resilience and adaptability to pandemic disruptions in the United States. Nat Med. 2024;30(8):2311‐2319. doi:10.1038/s41591-024-03103-6 38956198

[36] Metzl JM , Maybank A , De Maio F . Responding to the COVID‐19 pandemic: the need for a structurally competent health care system. JAMA. 2020;324(3):231‐232. doi:10.1001/jama.2020.9289 32496531

[37] Haggerty JL , Reid RJ , Freeman GK , Starfield BH , Adair CE , McKendry R . Continuity of care: a multidisciplinary review. BMJ. 2003;327(7425):1219‐1221. doi:10.1136/bmj.327.7425.1219 14630762 PMC274066

[38] Renbarger KM , Place JM , Schreiner M . The influence of four constructs of social support on pregnancy experiences in group prenatal care. Womens Health Rep (New Rochelle). 2021;2(1):154‐162. doi:10.1089/whr.2020.0113 34235502 PMC8243703

[39] Hill I , Cross‐Barnet C , Courtot B , Benatar S , Thornburgh S . What do women in Medicaid say about enhanced prenatal care? Findings from the national Strong Start evaluation. Birth. 2019;46(2):244‐252. doi:10.1111/birt.12431 31087393

[40] Mehay A , Motta GD , Hunter L , et al. What are the mechanisms of effect of group antenatal care? A systematic realist review and synthesis of the literature. BMC Pregnancy Childbirth. 2024;24(1):625. doi:10.1186/s12884-024-06792-6 39354405 PMC11446066

[41] Lewis JB , Cunningham SD , Shabanova V , et al. Group prenatal care and improved birth outcomes: results from a type 1 hybrid effectiveness‐implementation study. Prev Med. 2021;153:106853. doi:10.1016/j.ypmed.2021.106853 34678329

[42] National Committee for Quality Assurance . Prenatal and Postpartum Care. NCQA website. Accessed May 5, 2025. https://www.ncqa.org/report‐cards/health‐plans/state‐of‐health‐care‐quality‐report/prenatal‐and‐postpartum‐care‐ppc/

[43] Osterman MJK , Martin JA . Timing and adequacy of prenatal care in the United States, 2016. Natl Vital Stat Rep. 2018;67(3):1‐14. https://www.cdc.gov/nchs/data/nvsr/nvsr67/nvsr67_03.pdf 29874159

[44] Clapp MA , James KE , Kaimal AJ . Preconception insurance and initiation of prenatal care. J Perinatol. 2019;39(2):300‐306. doi:10.1038/s41372-018-0292-7 30546058

[45] Balk EM , Danilack VA , Cao W , et al. Televisits compared with in‐person visits for routine antenatal care: A systematic review. Obstet Gynecol. 2023;142(1):19‐29. doi:10.1097/AOG.0000000000005194 37290109

[46] Peahl AF , Smith RD , Moniz MH . Prenatal care redesign: creating flexible maternity care models through virtual care. Am J Obstet Gynecol. 2020;223(3):389.e1‐389.e10. doi:10.1016/j.ajog.2020.05.029 PMC7231494 32425200

[47] Lucas JW , Villarroel MA . Telemedicine use among adults: United States, 2021 . NCHS Data Brief, No. 445. National Center for Health Statistics. CDC website. October 2022. Accessed April 7, 2024. https://www.cdc.gov/nchs/products/databriefs/db445.htm 36255940

[48] Pflugeisen BM , McCarren C , Poore S , Carlile M , Schroeder R . Virtual visits: managing prenatal care with modern technology. MCN Am J Matern Child Nurs. 2016;41(1):24‐30. doi:10.1097/NMC.0000000000000199 26474477

[49] Wu KK , Lopez C , Nichols M . Virtual visits in prenatal care: An integrative review. J Midwifery Womens Health. 2022;67(1):39‐52. doi:10.1111/jmwh.13284 34767317

[50] Eruchalu CN , Pichardo MS , Bharadwaj M , et al. The expanding digital divide: digital health access inequities during the COVID‐19 pandemic in New York City. J Urban Health. 2021;98(2):183‐186. doi:10.1007/s11524-020-00508-9 33471281 PMC7816740

[51] Ghimire S , Martinez S , Hartvigsen G , Gerdes M . Virtual prenatal care: A systematic review of pregnant women's and healthcare professionals’ experiences, needs, and preferences for quality care. Int J Med Inform. 2023;170:104964. doi:10.1016/j.ijmedinf.2022.104964 36565547

[52] Marko KI , Ganju N , Krapf JM , et al. A mobile prenatal care app to reduce in person visits: prospective controlled trial. JMIR Mhealth Uhealth. 2019;7(5):e10520. doi:10.2196/10520 31042154 PMC6658303

[53] Duryea EL , Adhikari EH , Ambia A , Spong C , McIntire D , Nelson DB . Comparison between in person and audio‐only virtual prenatal visits and perinatal outcomes. JAMA Netw Open. 2021;4(4):e215854. doi:10.1001/jamanetworkopen.2021.5854 33852002 PMC8047732

[54] Palmer KR , Tanner M , Davies‐Tuck M , et al. Widespread implementation of a low‐cost telehealth service in the delivery of antenatal care during the COVID‐19 pandemic: an interrupted time‐series analysis. Lancet. 2021;398(10294):41‐52. doi:10.1016/S0140-6736(21)00668-1 34217399 PMC8248925

[55] Soffer MD , Sinnott C , Clapp MA , Bernstein SN . Impact of a hybrid model of prenatal care on the diagnosis of fetal growth restriction. Am J Perinatol. 2022;39(15):1605‐1613. doi:10.1055/a-1877-8478 35709745

[56] Sadiku F , Bucinca H , Talrich F , et al. Maternal satisfaction with group care: A systematic review. AJOG Glob Rep. 2023;4(1):100301. doi:10.1016/j.xagr.2023.100301 38318267 PMC10839533

[57] Peahl AF , Zahn CM , Turrentine M , et al. The Michigan plan for appropriate tailored healthcare in pregnancy prenatal care recommendations. Obstet Gynecol. 2021;138(4):593‐602. doi:10.1097/AOG.0000000000004531 34352810 PMC12101528

[58] Peahl AF , Howell JD . The evolution of prenatal care delivery guidelines in the United States. Am J Obstet Gynecol. 2021;224(4):339‐347. doi:10.1016/j.ajog.2020.12.016 33316276 PMC9745905

[59] Perry AF , Federico F , Huebner J . Telemedicine: Ensuring Safe, Equitable, Person‐Centered Virtual Care . Institute for Healthcare Improvement; 2021. https://www.ihi.org/resources/Pages/IHIWhitePapers/telemedicine‐safe‐equitable‐person‐centered‐virtual‐care.aspx

